# Erythroderma in Adult-Onset Still’s Disease Alleviated After Tocilizumab Therapy: A Case Report

**DOI:** 10.7759/cureus.60372

**Published:** 2024-05-15

**Authors:** Li-Na Zhang, Zheng Zhao, Juan-Juan Song, Bo-Zhi Lin, Tong Zhang

**Affiliations:** 1 Department of Rheumatology and Immunology, Peking University International Hospital, Beijing, CHN; 2 Department of Dermatology, Peking University International Hospital, Beijing, CHN; 3 Department of Nuclear Medicine, Peking University International Hospital, Beijing, CHN; 4 Department of Clinical Laboratory, Peking University International Hospital, Beijing, CHN; 5 Department of Pathology, Peking University International Hospital, Beijing, CHN

**Keywords:** primary biliary cholangitis, adult onset still's disease (aosd), exfoliative dermatitis, tocilizumab, erythroderma

## Abstract

Erythroderma, also known as exfoliative dermatitis, is a rarely reported atypical cutaneous manifestation of adult-onset Still’s disease (AOSD). We present the case of erythroderma in association with AOSD that was steroid dependent and responded to tocilizumab therapy. Skin rash, pruritis, and related laboratory findings were significantly improved upon the addition of tocilizumab, while prednisolone was successfully tapered to an ever-lowest maintenance level. To our knowledge, this is the first to report the sole therapeutic effect of tocilizumab in erythroderma related to AOSD.

## Introduction

Erythroderma, also known as exfoliative dermatitis, is an inflammatory disorder characterized by diffuse erythema and scaling. It may be a sign of a variety of underlying diseases, including infections, allergies, neoplasms, and autoimmune diseases [1]. When autoimmunity is involved, the rash frequently becomes steroid dependent and lacks effective treatment regimens. Erythroderma was reported among the atypical cutaneous manifestations of adult-onset Still’s disease (AOSD), which responded to tocilizumab in conjugation with high-dose steroids [2,3]. AOSD is a rare, systemic inflammatory disorder with a not completely understood etiology. Aberrant activation of the innate immune system and overproduction of several pro-inflammatory cytokines, including IL-6, are considered critical components in disease pathogenesis [4]. Four cardinal symptoms are fever, arthritis, evanescent rash, and leukocytosis. High dosages of corticosteroids are usually the first-line therapy. Despite this treatment, a large percentage of patients experience several flares with an evolution toward the chronic disease course or even life-threatening complications. Tocilizumab, a humanized anti-IL-6 receptor antibody, has verified its efficacy and tolerable safety in a variety of dermatological and rheumatological diseases, including AOSD, in which autoimmune and chronic inflammatory processes are deeply involved [4,5].

Herein, we report the case of a patient with a one-year history of AOSD and primary biliary cholangitis (PBC) who presented with persistent pruritic erythematous rash and scaling. A skin biopsy was performed, which was consistent with the diagnosis of erythroderma. Unlike previously reported cases in which high-dose steroids were added immediately to tocilizumab, our patient was taking a minimal maintenance dose of prednisolone during tocilizumab therapy. Thus, we are able to examine the sole therapeutic effect of tocilizumab in erythroderma related to AOSD. We fortunately collected a few valuable pictures before and after the administration of tocilizumab. This may provide some useful information for clinicians who are interested in refractory rash in AOSD.

## Case presentation

**Figure 1:**
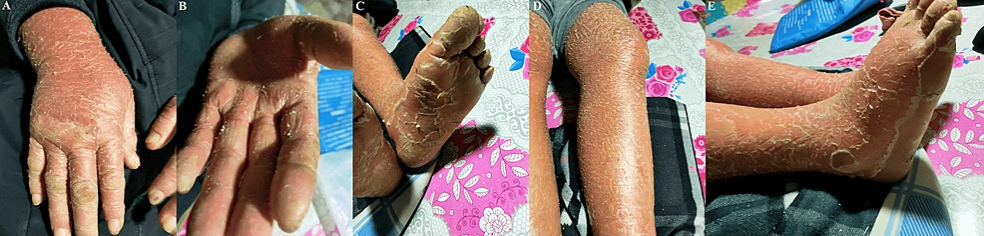
Photographs showing severe exfoliative dermatitis (A-E) Diffuse scaling involving the lower and upper extremities, accompanied by significant edema of the distal extremities.

A 55-year-old man with a one-year history of PBC presented with fever, rash, arthralgia, leukocytosis, hepatosplenomegaly, and hyperferritinemia. A PET-CT scan and bone marrow biopsy were performed to rule out malignancies and hemophagocytic lymphohistiocytosis. He was diagnosed with AOSD and was treated with prednisolone (initial dose: 80 mg/d) combined with azathioprine. His symptoms gradually relieved, with significant improvement in laboratory findings. However, he experienced a worsening relapse with fever and a new-onset rash when prednisolone was tapered to 15 mg/d. Physical examination revealed diffuse scaling on the body surface, which was suspicious for exfoliative dermatitis, also known as erythroderma. Tender edema of the distal extremities was apparent (Figure 1). Azathioprine was stopped, and the dosage of prednisolone was increased to 80 mg/d in conjunction with cyclophosphamide.

**Figure 2:**
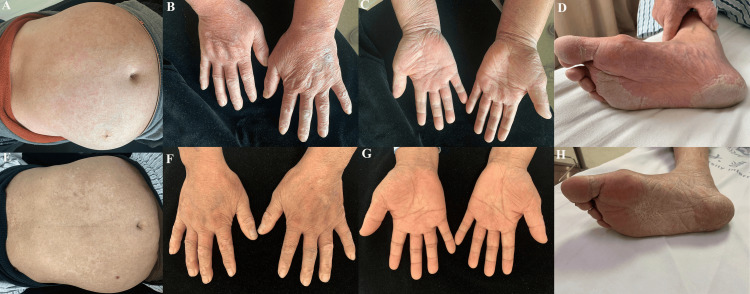
Photographs before (A-D) and eight weeks after (E-H) the application of tocilizumab Physical examination revealed an erythematous rash with dry, scaly skin bits on the trunk (A). Scaling on the hands and feet was also prominent (B, C, D). The erythematous rash on the trunk was dramatically relieved eight weeks later, leaving hypopigmentation but no scars (E), which was considered a sign of post-inflammatory change. Hypopigmentation was also noted on the back of the hands (F). Hyperkeratosis on the palms and plantar surfaces gradually resolved (G, H).

**Figure 3:**
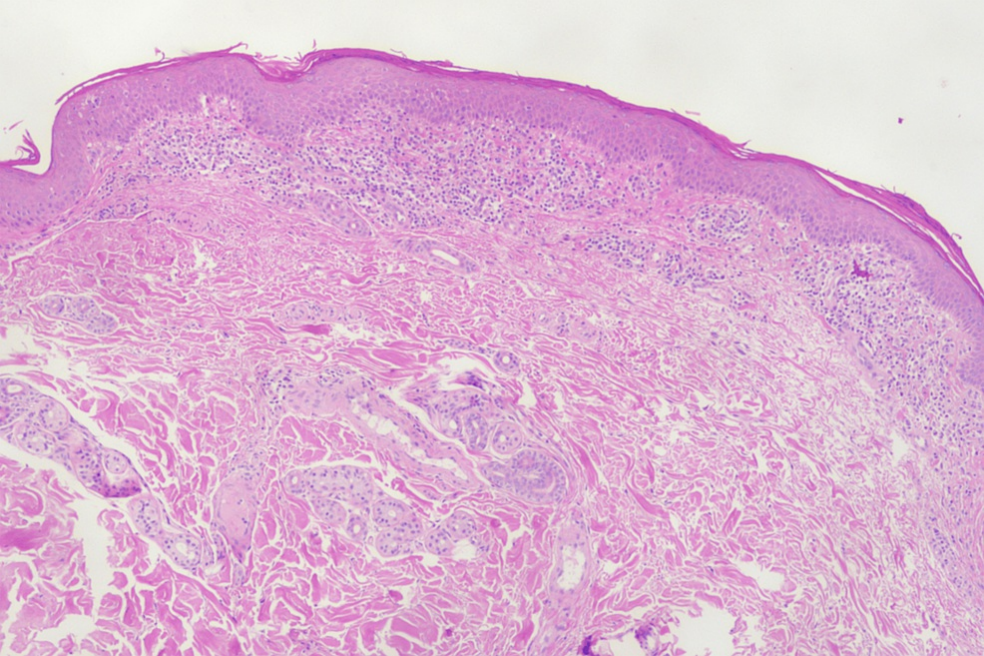
Skin biopsy of the abdomen before tocilizumab therapy Skin biopsy revealed linear aggregation of histiocytes and lymphocytes in the epidermis (H&E, ×40).

**Figure 4:**
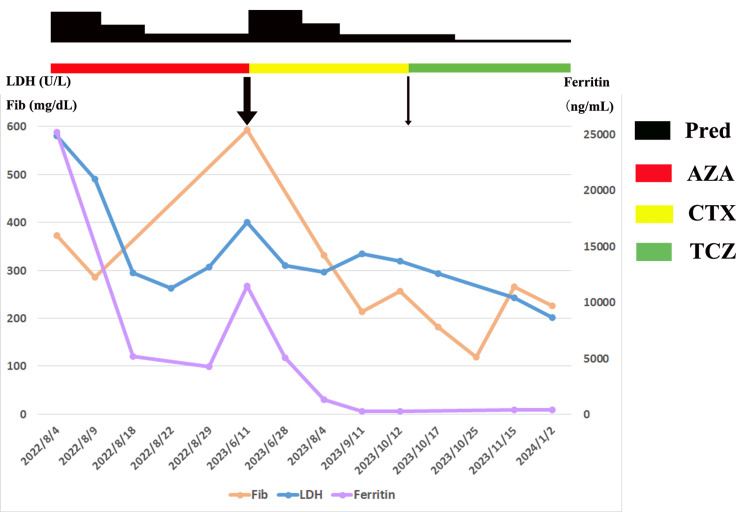
Curves of evolving laboratory values Markedly increased serum ferritin, LDH, and fibrinogen were noted at the onset of AOSD, which showed a dramatic response to steroid therapy. All these markers rose again when the patient experienced a severe relapse on a maintenance dose of prednisolone (15 mg/d) (thick arrow, corresponding to Figure 1). An increased dose of prednisolone (80 mg/d) resulted in decreased serum ferritin, LDH, and fibrinogen, consistent with partially alleviated skin rash. When oral prednisolone was slowly tapered to 15 mg/d, the patient revisited with a refractory pruritic rash (thin arrow, corresponding to Figure 2A, 2B, 2C, 2D). Serum ferritin, LDH, and fibrinogen levels tested at the same time were not as high as those at the previous relapse (thick arrow). Notably, the addition of tocilizumab significantly decreased fibrinogen and LDH levels over time. AZA, azathioprine; CTX, cyclophosphamide; LDH, lactate dehydrogenase; Pred, prednisolone; TCZ, tocilizumab

The patient’s skin rash was partially alleviated upon the addition of prednisolone. However, when oral prednisolone was slowly tapered to 15 mg/d, the patient revisited the complaint of refractory pruritic rash. Physical examination showed an erythematous rash with dry, scaly skin bits on the trunk (Figure 2A). Scaling on the hands and feet was also prominent (Figure 2B, 2C, 2D). A skin biopsy of the abdomen revealed a linear aggregation of inflammatory cells in the epidermis (Figure 3). These features were consistent with erythroderma [1]. He was also notable for Cushing’s syndrome, obesity, and steroid-induced cataracts. To avoid frequent relapses and increased steroid intake, tocilizumab (8 mg/kg) was applied at four-week intervals in combination with a maintenance dose of prednisolone (15 mg/d). Cyclophosphamide was discontinued in the meantime. The erythematous rash on the trunk was dramatically relieved eight weeks later (Figure 2E). Hyperkeratosis on the hands and plantar surfaces was also resolved (Figure 2F, 2G, 2H). Pruritus greatly improved during the subsequent weeks. Moreover, the prednisolone was successfully tapered to an ever-lowest dose of 7.5 mg/d without any symptoms. Curves of evolving laboratory values, including serum ferritin, lactate dehydrogenase (LDH), and fibrinogen, are presented in Figure 4.

## Discussion

Erythroderma, also known as exfoliative dermatitis, is a rarely reported atypical cutaneous manifestation of AOSD [1]. In previously reported cases, erythroderma in AOSD was successfully treated with tocilizumab in conjugation with high-dose steroids [2,3]. We used a maintenance dose of prednisolone during tocilizumab therapy because the patient’s rash was relatively mild compared to the previous reports. Thus, it was unnecessary to initiate high-dose steroids immediately. Disease activity can be reflected by the severity of the skin rash and laboratory findings, such as serum ferritin, LDH, and fibrinogen levels [6]. In fact, the patient had experienced a much more severe relapse in the first cycle of steroid therapy when prednisolone was tapered to 15 mg/d (Figure 1; Figure 4, thick arrow). An increased dose of prednisolone was applied, which partially alleviated his symptoms. At the end of the second cycle of steroid therapy (prednisolone 15 mg/d), when the patient came back with refractory rash, his skin rash and laboratory markers were milder (Figure 2A, 2B, 2C, 2D; Figure 4, thin arrow), compared to the former (Figure 1; Figure 4, thick arrow). Nevertheless, milder manifestations should not be overlooked, since the patient’s rash persisted and he apparently became steroid dependent. He developed Cushing’s syndrome and had difficulty tapering off prednisolone, despite the application of conventional steroid-sparing agents like azathioprine and cyclophosphamide. Searching for effective steroid-sparing drugs is urgent to prevent long-term complications of high-dose steroid intake and potential relapses.

The patient’s rash was significantly alleviated upon the addition of tocilizumab to a maintenance dose of prednisolone (Figure 2E, 2F, 2G, 2H). Moreover, his pruritus greatly improved during the subsequent weeks, and his prednisolone was tapered to an ever-lowest dose of 7.5 mg/d. This provided an excellent model to understand the sole therapeutic effect of tocilizumab in erythroderma related to AOSD. Unlike previous reports in which high-dose steroids were applied together with tocilizumab, the influence of steroids was eliminated in the present case. It is noteworthy that fibrinogen and LDH levels decreased after tocilizumab therapy, while serum ferritin remained stable (Figure 4). We speculated that fibrinogen and LDH levels, as well as skin changes, served as more sensitive markers of disease activity compared to serum ferritin, a traditional marker in AOSD. Skin findings may be an important early clue to a severe relapse and aid in prompt recognition and treatment of this potentially life-threatening disease.

Hypersensitivity to azathioprine should be considered in the differential diagnosis of erythroderma in our patient. Nevertheless, clinical and pathological features differed between erythroderma and hypersensitivity to azathioprine. Cutaneous manifestation of azathioprine hypersensitivity syndrome is a rare side effect and typically occurs early in the initiation of therapy [7]. In the present case, erythroderma occurred more than six months after the application of azathioprine, which lasted for four months after the discontinuation of azathioprine. Furthermore, biopsy results were consistent with neutrophilic dermatosis for azathioprine hypersensitivity syndrome. In contrast, a skin biopsy of the present case showed epidermal infiltration consisting of histiocytes and lymphocytes (Figure 3). Erythroderma in the present case could be attributed to autoimmune disturbances associated with AOSD rather than hypersensitivity.

Tocilizumab, a humanized anti-IL-6 receptor antagonist, proved to be an effective drug with a favorable overall safety profile for curing a variety of dermatological and rheumatological diseases, including AOSD [5,8]. The therapeutic effect of tocilizumab was associated with inhibition of IL-6, a well-known pro-inflammatory cytokine, which was markedly elevated and correlated with systemic symptoms in AOSD [9]. Herein, we displayed a rare case of erythroderma, a severe cutaneous complication of AOSD. The patient showed an excellent response to tocilizumab after several unsuccessful trials of conventional steroid-sparing agents. The application of tocilizumab resulted in dramatic resolution of cutaneous complaints, including refractory exfoliative dermatitis and persistent pruritis. The drug also improved laboratory markers reflecting disease activities (Figure 4). Moreover, the prednisolone was successfully tapered to an ever-lowest maintenance dose of 7.5 mg/d without any symptoms. However, it is noteworthy that, despite the verified tolerable safety of tocilizumab in many rheumatic diseases, rare cases of tocilizumab-induced erythroderma have been described [10,11]. Thus, extra watchfulness is warranted in patients with a high risk of severe infection and allergic reactions.

## Conclusions

In this case, tocilizumab showed a clear benefit for treating erythroderma related to AOSD with a significant steroid-sparing effect. To our knowledge, this is the first to report the sole therapeutic effect of tocilizumab in erythroderma in the setting of AOSD. We fortunately collected a few valuable pictures before and after the administration of tocilizumab. This may provide some useful information for clinicians who are interested in the topic. Our case suggested that tocilizumab might be an effective therapeutic agent in the treatment of refractory rash in AOSD. However, watchfulness is warranted in patients with a high risk of severe infection and allergic reactions.
